# Crystal structure and Hirshfeld surface analysis of 5-methyl-1,2,4-triazolo[1,5-*a*]pyrimidine

**DOI:** 10.1107/S2056989018016225

**Published:** 2018-11-22

**Authors:** Sanae Lahmidi, Nada Kheira Sebbar, Tuncer Hökelek, Karim Chkirate, Joel T. Mague, El Mokhtar Essassi

**Affiliations:** a Laboratoire de Chimie Organique Hétérocyclique URAC 21, Pôle de Compétence Pharmacochimie, Av. Ibn Battouta, BP 1014, Faculté des Sciences, Université Mohammed V, Rabat, Morocco; b Laboratoire de Chimie Bioorganique Appliquée, Faculté des Sciences, Université Ibn Zohr, Agadir, Morocco; c Department of Physics, Hacettepe University, 06800 Beytepe, Ankara, Turkey; d Department of Chemistry, Tulane University, New Orleans, LA 70118, USA

**Keywords:** crystal structure, triazole, pyrimidine, hydrogen bond, π⋯π-stacking, Hirshfeld surface analysis

## Abstract

The nine-membered ring system of the title compound, C_6_H_6_N_4_, is essentially planar. In the crystal, mol­ecules are linked *via* C—H_Trz_⋯N_Trz_ and C—H_Pyrm_⋯N_Trz_ (Trz = triazole and Pyrm = pyrimidine) hydrogen bonds together with weaker C—H_Pyrm_⋯N_Pyrm_ hydrogen bonds to form layers parallel to (02). The layers are further connected by π–π-stacking inter­actions between the nine-membered ring system [centroid–centroid = 3.7910 (8) Å], forming oblique stacks along the *a*-axis direction. The Hirshfeld surface analysis of the crystal structure indicates that the most important contributions for the crystal packing are from H⋯N/N⋯H (40.1%), H⋯H (35.3%), H⋯C/C⋯H (9.5%), N⋯C/C⋯N (9.0%), N⋯N (3.1%) and C⋯C (3.0%) inter­actions and that hydrogen-bonding and van der Waals inter­actions are the dominant inter­actions in the crystal packing. No significant C—H⋯π inter­actions are observed.

## Abstract 2

The nine-membered ring system of the title compound is essentially planar. In the crystal, mol­ecules are linked *via* C—H_Trz_⋯N_Trz_ and C—H_Pyrm_⋯N_Trz_ (Trz = triazole and Pyrm = pyrimidine) hydrogen bonds together with weaker C—H_Pyrm_⋯N_Pyrm_ hydrogen bonds to form layers parallel to (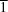
02). The layers are further connected by π–π-stacking inter­actions between the nine-membered ring system, forming oblique stacks along the *a*-axis direction.

## Chemical context   

In recent years, much attention has been paid to the development of new methods for the synthesis and investigation of biological and pharmacological properties of [1,2,4]triazolo[1,5-*a*]pyrimidine derivatives (Chebanov *et al.*, 2010 ▸; Lahmidi *et al.*, 2016*a* ▸,*b* ▸, 2018 ▸; Sedash *et al.*, 2012 ▸). Thus, these compounds have also received successful applications for the preparation of new poly-condensed heterocycles (Beck *et al.*, 2011 ▸). Among the various classes of nitro­gen-containing heterocyclic compounds such as triazolo­pyrimidine derivatives display a broad spectrum of biological activities, including anti-inflammatory (Ashour *et al.*, 2013 ▸), anti­cancer (Hoffmann *et al.*, 2017 ▸) and anti­bacterial (Mabkhot *et al.*, 2016 ▸) activities. In a continuation of our research on the elaboration of new methods for the synthesis of various heterocyclic systems, we investigated the reaction of bis­(2-chloro­eth­yl)amine hydro­chloride with ethyl 2-(5-methyl-1-1,2,4-triazolo[1,5-*a*]pyrimidin-7-yl)acetate under phase-transfer catalysis conditions using tetra-*n*-butyl ammonium­bromide (TBAB) as catalyst and potassium carbonate as base to afford the title compound, 5-methyl-1,2,4-triazolo[1,5-*a*]pyrimidine, (I). We report herein its mol­ecular and crystal structures along with the results of a Hirshfeld surface analysis.
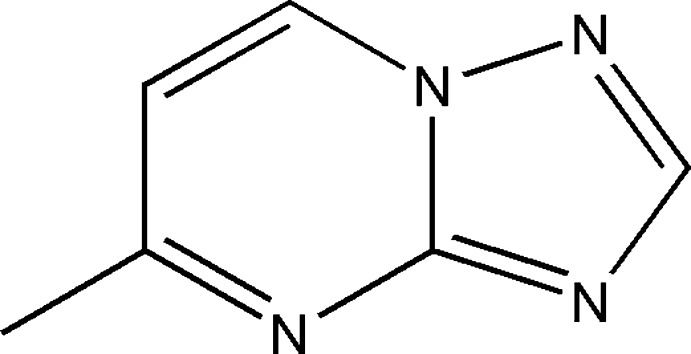


## Structural commentary   

**Figure 1:**
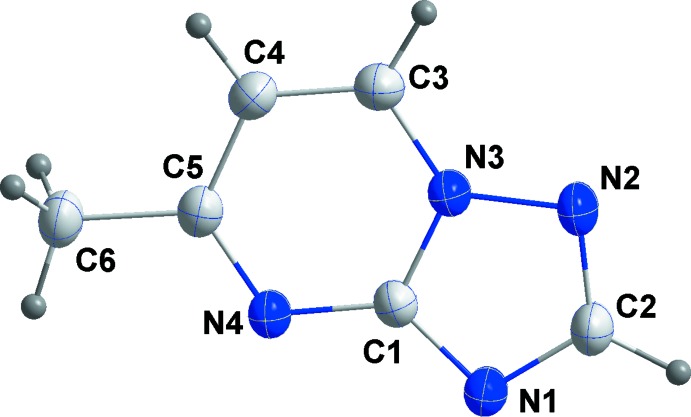
The title mol­ecule with the atom-labelling scheme and 50% probability ellipsoids.

In the title compound (Fig. 1 ▸), the nine-membered ring is planar to within 0.004 (1) Å (for atom C5), and the r.m.s. deviation of the fitted atoms is 0.009 Å. Methyl atom C6 is displaced by 0.032 (1) Å from the ring system.

## Supra­molecular features   

**Figure 2:**
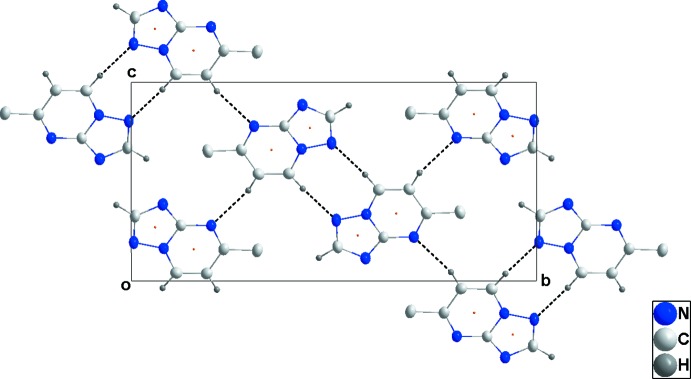
The packing viewed along the *a*-axis direction giving a plan view of the layers. C—H⋯N hydrogen bonds are shown as black dashed lines and the orange dots mark the π–π stacking inter­actions.

**Figure 3:**
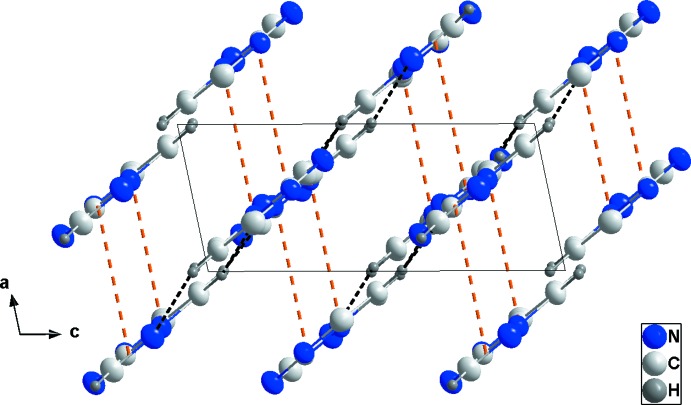
Packing seen along the *b*-axis direction giving a side view of the layers. Hydrogen bonds are depicted as in Fig. 2 ▸ and the π-stacking inter­actions are shown as orange dashed lines.

In the crystal, C—H_Trz_⋯N_Trz_ and C—H_Pyrm_⋯N_Trz_ (Trz = triazole and Pyrm = pyrimidine) hydrogen bonds (Table 1 ▸), together with weaker C—H_Pyrm_⋯N_Pyrm_ hydrogen bonds, link the mol­ecules, forming layers parallel to (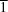
02) (Fig. 2 ▸). The layers are further connected by π–π-stacking inter­actions between the nine-membered rings [centroid–centroid distance = 3.7910 (8) Å], forming oblique stacks along the *a*-axis direction (Fig. 3 ▸). No significant C—H ⋯ π inter­actions are observed.

## Hirshfeld surface analysis   

**Figure 4:**
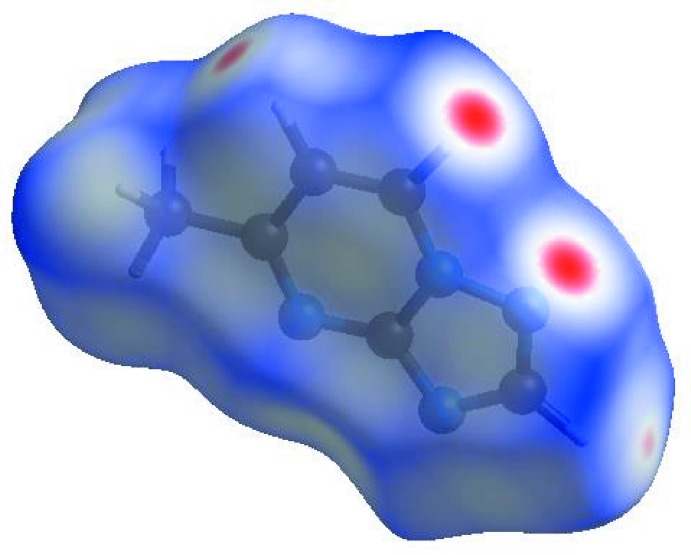
View of the three-dimensional Hirshfeld surface of the title compound plotted over *d*_norm_ in the range −0.1566 to 1.0057 a.u.

**Figure 5:**
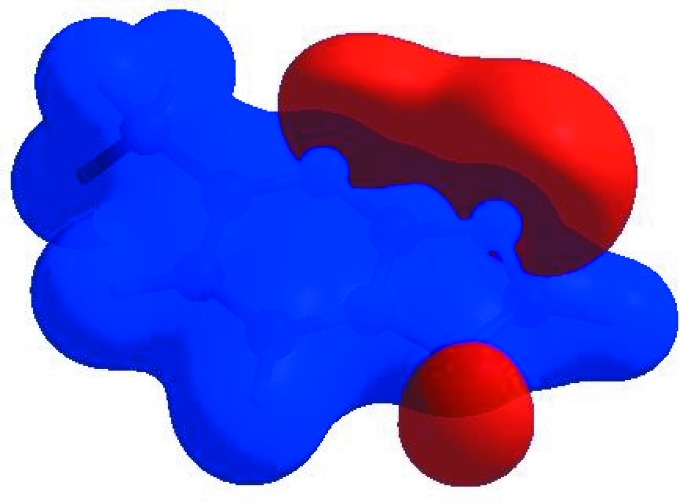
View of the three-dimensional Hirshfeld surface of the title compound plotted over electrostatic potential energy in the range −0.0500 to 0.0500 a.u. using the STO-3 G basis set at the Hartree–Fock level of theory. Hydrogen-bond donors and acceptors are shown as blue and red regions around the atoms corresponding to positive and negative potentials, respectively.

**Figure 6:**
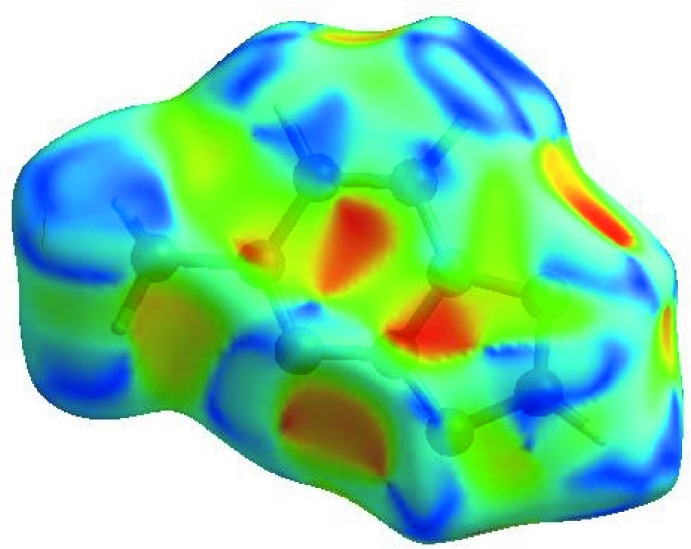
Hirshfeld surface of the title compound plotted over shape-index.

In order to visualize the inter­molecular inter­actions in the crystal of the title compound, a Hirshfeld surface (HS) analysis (Hirshfeld, 1977 ▸; Spackman & Jayatilaka, 2009 ▸) was carried out using *CrystalExplorer17.5* (Turner *et al.*, 2017 ▸). In the HS plotted over *d*_norm_ (Fig. 4 ▸), the white surface indicates contacts with distances equal to the sum of the van der Waals radii, and the red and blue colours indicate distances shorter (in close contact) or longer (distinct contact), respectively, than the van der Waals radii (Venkatesan *et al.*, 2016 ▸). The bright-red spots appearing near N2 and hydrogen atoms H2, H3 and H4 indicate their roles as the respective donors and/or acceptors in the dominant C—H⋯N hydrogen bonds; they also appear as blue and red regions corresponding to positive and negative potentials on the HS mapped over electrostatic potential (Spackman *et al.*, 2008 ▸; Jayatilaka *et al.*, 2005 ▸) as shown in Fig. 5 ▸. The blue regions indicate positive electrostatic potential (hydrogen-bond donors), while the red regions indicate negative electrostatic potential (hydrogen-bond acceptors). The shape-index of the HS is a tool to visualize π–π stacking by the presence of adjacent red and blue triangles; if there are no adjacent red and/or blue triangles, then there are no π–π inter­actions. Fig. 6 ▸ clearly suggest that there are π–π inter­actions present in the crystal structure of (I).

**Figure 7:**
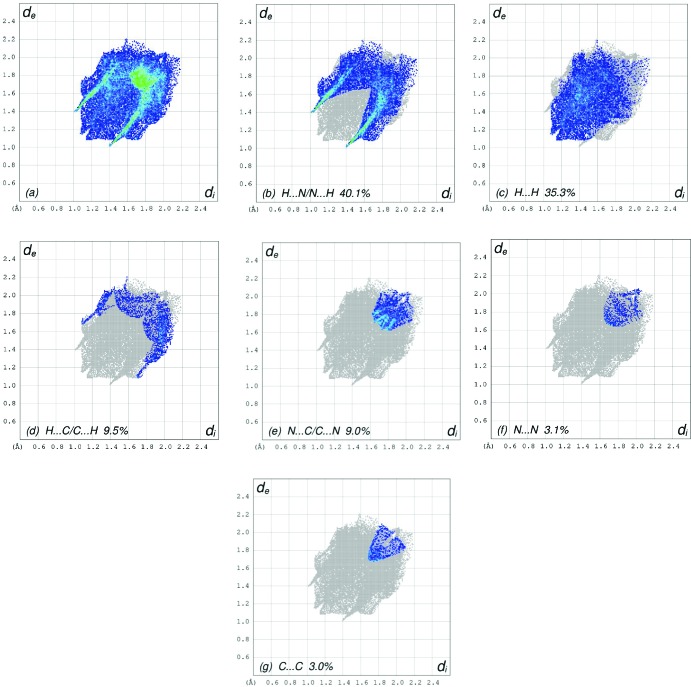
The full two-dimensional fingerprint plots for the title compound, showing (*a*) all inter­actions, and delineated into (*b*) H⋯N/N⋯H, (*c*) H⋯H, (*d*) H⋯C/C⋯H, (*e*) N⋯C/C⋯N, (*f*) N⋯N and (*g*) C⋯C inter­actions. The *d*_i_ and *d*_e_ values are the closest inter­nal and external distances (in Å) from given points on the Hirshfeld surface contacts.

The overall two-dimensional fingerprint plot, Fig. 7 ▸(*a*), and those delineated into H⋯N/N⋯H, H⋯H, H⋯C/C⋯H, N⋯C/C⋯N, N⋯N and C⋯C contacts (McKinnon *et al.*, 2007 ▸) are illustrated in Fig. 7 ▸(*b*)–(*g*), respectively, together with their relative contributions to the Hirshfeld surface. The most important inter­action is H⋯N/N⋯H, contributing 40.1% to the overall crystal packing, which is reflected in Fig. 7 ▸(*b*) as a pair of characteristic wings with the tips at *d*_e_ + *d*_i_ = 2.40 Å arising from the C—H⋯N hydrogen bonds (Table 1 ▸) as well as from the H⋯N/N⋯H contacts (Table 3 ▸). The split thin and thick pair of wings with the tips at *d*_e_ + *d*_i_ ∼2.23 Å in Fig. 7 ▸(*c*), arise from the short inter­atomic H⋯H contacts, which make a 35.3% contribution to the HS and are seen as widely scattered points of high density arising from the large hydrogen content of the mol­ecule. In the absence of C—H⋯π inter­actions, the pair of wings in the fingerprint plot delineated into H⋯C/C⋯H contacts (9.5% contribution to the HS) have a nearly symmetrical distribution of points, Fig. 7 ▸(*d*), with the tips at *d*_e_ + *d*_i_ ∼2.77 Å. The N⋯C/C⋯N [Fig. 7 ▸(*e*)] and N⋯N [Fig. 7 ▸(*f*)] contacts make contributions of 9.0 and 3.1%, respectively, to the HS and have widely scattered distributions of points. Finally, the C⋯C [Fig. 7 ▸(*g*)] contacts (3.0% contribution to the HS) have a symmetrical distribution of points, with the tip at *d*_e_ = *d*_i_ = 1.69 Å.

**Figure 8:**
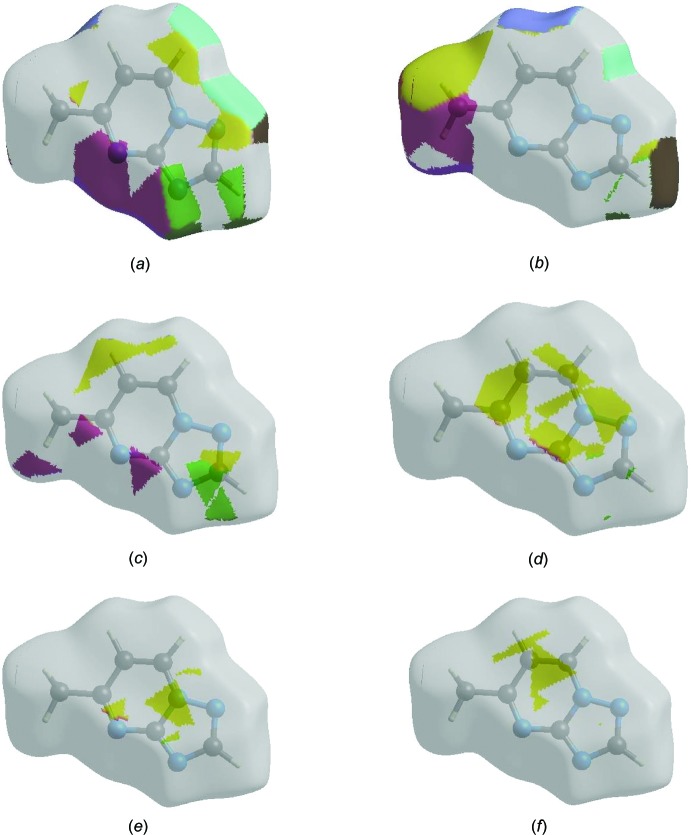
The Hirshfeld surface representations with the function *d*_norm_ plotted onto the surface for (*a*) H⋯N/N⋯H, (*b*) H⋯H, (*c*) H⋯C/C⋯H, (*d*) N⋯C/C⋯N, (*e*) N⋯N and (*f*) C⋯C inter­actions.

The Hirshfeld surface representations with the function *d*_norm_ plotted onto the surface are shown for the H⋯N/N⋯H, H⋯H, H⋯C/C⋯H, N ⋯ C/C⋯N, N⋯N and C⋯C inter­actions in Fig. 8 ▸(*a*)–(*f*), respectively.

The Hirshfeld surface analysis confirms the importance of H-atom contacts in establishing the packing. The large number of H⋯N/N⋯H, H⋯H and H⋯C/C⋯H inter­actions suggest that van der Waals inter­actions and hydrogen bonding play the major roles in the crystal packing (Hathwar *et al.*, 2015 ▸).

## Database survey   

Two structures have previously been reported in which the title compound, (I), is present as a ligand (*L*), namely [Fe(*L*)_2_(SCN)_2_(H_2_O)_2_] (Bigini Cingi *et al.*, 1986 ▸) and [Cu(μ-*L*)_2_(SCN)]_*n*_ (Cornelissen *et al.*, 1989 ▸), but to the best of our knowledge, the mol­ecule itself has not previously been structurally characterized.

## Synthesis and crystallization   

To a solution of ethyl-2-{5-methyl-1-[1,2,4]triazolo[1,5-a]pyrimidin-7-yl}acetate (1.00 g, 4.5 mmol) in DMF (25 ml) was added 2eq of bis­(2-chloro­eth­yl)amine hydro­chloride (1.61g, 9 mmol), potassium carbonate (1.37 g, 9.9 mmol) and a catalytic amount of tetra-*n*-but­ylammonium bromide. The mixture was stirred at 353.15 K for 24 h. The solution was filtered and the solvent was removed under reduced pressure. The residue obtained was dissolved in di­chloro­methane and purified by column chromatography (EtOAc/Hexane, 1:9 *v*:*v*). The title compound was obtained as colourless crystals in 40% yield.

## Refinement   

Crystal data, data collection and structure refinement details are summarized in Table 2 ▸. H atoms were located in a difference Fourier map and were freely refined.

## Supplementary Material

Crystal structure: contains datablock(s) I, global. DOI: 10.1107/S2056989018016225/lh5886sup1.cif

Structure factors: contains datablock(s) I. DOI: 10.1107/S2056989018016225/lh5886Isup2.hkl

Supporting information file. DOI: 10.1107/S2056989018016225/lh5886Isup3.cdx

Supporting information file. DOI: 10.1107/S2056989018016225/lh5886Isup4.cml

CCDC reference: 1879279

Additional supporting information:  crystallographic information; 3D view; checkCIF report

## Figures and Tables

**Table 1:** Hydrogen-bond geometry (Å, °)

*D*—H⋯*A*	*D*—H	H⋯*A*	*D*⋯*A*	*D*—H⋯*A*
C2—H2⋯N1^i^	1.016 (17)	2.550 (19)	3.4052 (18)	141.5 (13)
C3—H3⋯N2^vi^	0.979 (18)	2.525 (18)	3.4822 (18)	165.8 (13)
C4—H4⋯N4^viii^	0.946 (19)	2.642 (19)	3.5677 (17)	165.9 (14)

Symmetry codes: (i) 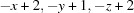; (vi) 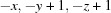; (viii) 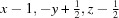.

**Table 2:** Selected interatomic distances (Å)

N1⋯C2^i^	3.4051 (19)	C1⋯C4^iii^	3.5667 (19)
N2⋯C2^ii^	3.385 (2)	C2⋯C6^vii^	3.5715 (18)
N3⋯C3^iii^	3.4163 (19)	C2⋯C2^i^	3.595 (2)
N4⋯C5^iii^	3.4314 (17)	C4⋯C5^ii^	3.4986 (19)
N4⋯C4^iii^	3.4177 (19)	C1⋯H6*B* ^iv^	2.94 (3)
N1⋯H6*B* ^iv^	2.85 (2)	C6⋯H6*C* ^iii^	2.98 (3)
N1⋯H2^i^	2.553 (18)	H2⋯C6^vii^	2.773 (16)
N1⋯H6*C* ^v^	2.86 (3)	H2⋯H6*B* ^vii^	2.58 (3)
N2⋯H3^vi^	2.525 (18)	H2⋯H6*C* ^vii^	2.48 (3)
N4⋯H4^v^	2.641 (18)	H6*A*⋯H4^v^	2.59 (3)
N4⋯H6*B* ^iv^	2.84 (3)	H6*B*⋯H6*C* ^iii^	2.47 (4)
C1⋯C3^iii^	3.4166 (19)		

Symmetry codes: (i) 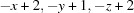; (ii) 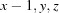; (iii) 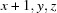; (iv) 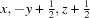; (v) 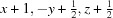; (vi) 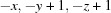; (vii) 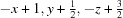.

**Table 3:** Experimental details

Crystal data	
Chemical formula	C_6_H_6_N_4_
*M*_r_	134.15
Crystal system, space group	Monoclinic, *P*2_1_/*c*
Temperature (K)	150
*a*, *b*, *c* (Å)	3.7910 (2), 18.0092 (10), 9.0069 (5)
β (°)	101.704 (2)
*V* (Å^3^)	602.14 (6)
*Z*	4
Radiation type	Cu *K*α
μ (mm^−1^)	0.82
Crystal size (mm)	0.29 × 0.18 × 0.13

Data collection	
Diffractometer	Bruker D8 VENTURE PHOTON 100 CMOS
Absorption correction	Multi-scan (*SADABS*; Krause *et al.*, 2015 ▸)
*T*_min_, *T*_max_	0.67, 0.90
No. of measured, independent and observed [*I* > 2σ(*I*)] reflections	4567, 1205, 1102
*R*_int_	0.074
(sin θ/λ)_max_ (Å^−1^)	0.626

Refinement	
*R*[*F* ^2^ > 2σ(*F* ^2^)], *wR*(*F* ^2^), *S*	0.044, 0.113, 1.10
No. of reflections	1205
No. of parameters	116
H-atom treatment	All H-atom parameters refined
Δρ_max_, Δρ_min_ (e Å^−3^)	0.20, −0.20

Computer programs: *APEX3* and *SAINT* (Bruker, 2016 ▸), *SHELXT* (Sheldrick, 2015*a* ▸), *SHELXL2018* (Sheldrick, 2015*b* ▸), *DIAMOND* (Brandenburg & Putz, 2012 ▸) and *SHELXTL* (Sheldrick, 2008 ▸).

## supplementary crystallographic information

### Crystal data

**Table:**

C_6_H_6_N_4_	*F*(000) = 280
*M**_r_* = 134.15	*D*_x_ = 1.480 Mg m^−^^3^
Monoclinic, *P*2_1_/*c*	Cu *K*α radiation, λ = 1.54178 Å
*a* = 3.7910 (2) Å	Cell parameters from 3969 reflections
*b* = 18.0092 (10) Å	θ = 4.9–74.7°
*c* = 9.0069 (5) Å	µ = 0.82 mm^−^^1^
β = 101.704 (2)°	*T* = 150 K
*V* = 602.14 (6) Å^3^	Column, colourless
*Z* = 4	0.29 × 0.18 × 0.13 mm

### Data collection

**Table:**

Bruker D8 VENTURE PHOTON 100 CMOS diffractometer	1205 independent reflections
Radiation source: INCOATEC IµS micro-focus source	1102 reflections with *I* > 2σ(*I*)
Mirror monochromator	*R*_int_ = 0.074
Detector resolution: 10.4167 pixels mm^-1^	θ_max_ = 74.7°, θ_min_ = 4.9°
ω scans	*h* = −4→4
Absorption correction: multi-scan (*SADABS*; Krause *et al.*, 2015)	*k* = −22→21
*T*_min_ = 0.67, *T*_max_ = 0.90	*l* = −11→10
4567 measured reflections	

### Refinement

**Table:**

Refinement on *F*^2^	Secondary atom site location: difference Fourier map
Least-squares matrix: full	Hydrogen site location: difference Fourier map
*R*[*F*^2^ > 2σ(*F*^2^)] = 0.044	All H-atom parameters refined
*wR*(*F*^2^) = 0.113	*w* = 1/[σ^2^(*F*_o_^2^) + (0.0458*P*)^2^ + 0.1643*P*] where *P* = (*F*_o_^2^ + 2*F*_c_^2^)/3
*S* = 1.10	(Δ/σ)_max_ < 0.001
1205 reflections	Δρ_max_ = 0.20 e Å^−^^3^
116 parameters	Δρ_min_ = −0.20 e Å^−^^3^
0 restraints	Extinction correction: *SHELXL2018* (Sheldrick, 2015*b*), Fc^*^=kFc[1+0.001xFc^2^λ^3^/sin(2θ)]^-1/4^
Primary atom site location: structure-invariant direct methods	Extinction coefficient: 0.021 (4)

### Special details

**Table:**

Geometry. All esds (except the esd in the dihedral angle between two l.s. planes) are estimated using the full covariance matrix. The cell esds are taken into account individually in the estimation of esds in distances, angles and torsion angles; correlations between esds in cell parameters are only used when they are defined by crystal symmetry. An approximate (isotropic) treatment of cell esds is used for estimating esds involving l.s. planes.
Refinement. Refinement of F^2^ against ALL reflections. The weighted R-factor wR and goodness of fit S are based on F^2^, conventional R-factors R are based on F, with F set to zero for negative F^2^. The threshold expression of F^2^ > 2sigma(F^2^) is used only for calculating R-factors(gt) etc. and is not relevant to the choice of reflections for refinement. R-factors based on F^2^ are statistically about twice as large as those based on F, and R- factors based on ALL data will be even larger.

### Fractional atomic coordinates and isotropic or equivalent isotropic displacement parameters (Å^2^)

**Table:**

	*x*	*y*	*z*	*U*_iso_*/*U*_eq_	
N1	0.7561 (3)	0.42236 (6)	0.88570 (13)	0.0322 (3)	
N2	0.4407 (3)	0.49411 (6)	0.69436 (15)	0.0341 (3)	
N3	0.3764 (3)	0.42019 (6)	0.66430 (13)	0.0281 (3)	
N4	0.5532 (3)	0.30294 (6)	0.77958 (13)	0.0277 (3)	
C1	0.5676 (3)	0.37780 (7)	0.78059 (15)	0.0269 (3)	
C2	0.6678 (4)	0.49082 (7)	0.82759 (17)	0.0340 (4)	
H2	0.776 (5)	0.5371 (9)	0.883 (2)	0.037 (4)*	
C3	0.1587 (4)	0.38918 (7)	0.54119 (15)	0.0313 (3)	
H3	0.025 (5)	0.4230 (9)	0.465 (2)	0.037 (4)*	
C4	0.1409 (4)	0.31397 (7)	0.53851 (16)	0.0302 (3)	
H4	−0.006 (5)	0.2889 (10)	0.456 (2)	0.039 (4)*	
C5	0.3442 (3)	0.27179 (7)	0.66009 (15)	0.0279 (3)	
C6	0.3279 (4)	0.18893 (7)	0.65408 (19)	0.0344 (4)	
H6A	0.462 (6)	0.1656 (13)	0.749 (3)	0.066 (6)*	
H6B	0.431 (6)	0.1705 (12)	0.576 (3)	0.071 (7)*	
H6C	0.095 (7)	0.1716 (12)	0.629 (3)	0.073 (7)*	

### Atomic displacement parameters (Å^2^)

**Table:**

	*U*^11^	*U*^22^	*U*^33^	*U*^12^	*U*^13^	*U*^23^
N1	0.0381 (6)	0.0233 (5)	0.0321 (6)	−0.0020 (4)	0.0000 (5)	−0.0010 (4)
N2	0.0434 (7)	0.0197 (5)	0.0367 (6)	−0.0011 (4)	0.0026 (5)	0.0000 (4)
N3	0.0324 (6)	0.0222 (5)	0.0281 (6)	−0.0001 (4)	0.0024 (5)	0.0005 (4)
N4	0.0311 (6)	0.0221 (5)	0.0289 (6)	−0.0004 (4)	0.0040 (5)	−0.0003 (4)
C1	0.0297 (6)	0.0224 (6)	0.0280 (7)	0.0001 (4)	0.0041 (5)	0.0011 (4)
C2	0.0414 (8)	0.0221 (6)	0.0360 (8)	−0.0023 (5)	0.0020 (6)	−0.0019 (5)
C3	0.0337 (7)	0.0304 (7)	0.0281 (7)	0.0005 (5)	0.0020 (5)	0.0005 (5)
C4	0.0319 (7)	0.0289 (7)	0.0284 (7)	−0.0030 (5)	0.0026 (5)	−0.0029 (5)
C5	0.0284 (6)	0.0250 (6)	0.0308 (7)	−0.0016 (5)	0.0074 (5)	−0.0022 (5)
C6	0.0386 (8)	0.0244 (7)	0.0393 (8)	−0.0022 (5)	0.0058 (7)	−0.0043 (5)

### Geometric parameters (Å, º)

**Table:**

N1—C1	1.3329 (17)	C3—C4	1.3560 (18)
N1—C2	1.3545 (17)	C3—H3	0.979 (18)
N2—C2	1.329 (2)	C4—C5	1.4241 (19)
N2—N3	1.3703 (15)	C4—H4	0.946 (19)
N3—C3	1.3607 (17)	C5—C6	1.4940 (18)
N3—C1	1.3775 (17)	C6—H6A	1.00 (2)
N4—C5	1.3245 (17)	C6—H6B	0.94 (3)
N4—C1	1.3492 (17)	C6—H6C	0.92 (3)
C2—H2	1.016 (17)		
			
N1···C2^i^	3.4051 (19)	C1···C4^iii^	3.5667 (19)
N2···C2^ii^	3.385 (2)	C2···C6^vii^	3.5715 (18)
N3···C3^iii^	3.4163 (19)	C2···C2^i^	3.595 (2)
N4···C5^iii^	3.4314 (17)	C4···C5^ii^	3.4986 (19)
N4···C4^iii^	3.4177 (19)	C1···H6B^iv^	2.94 (3)
N1···H6B^iv^	2.85 (2)	C6···H6C^iii^	2.98 (3)
N1···H2^i^	2.553 (18)	H2···C6^vii^	2.773 (16)
N1···H6C^v^	2.86 (3)	H2···H6B^vii^	2.58 (3)
N2···H3^vi^	2.525 (18)	H2···H6C^vii^	2.48 (3)
N4···H4^v^	2.641 (18)	H6A···H4^v^	2.59 (3)
N4···H6B^iv^	2.84 (3)	H6B···H6C^iii^	2.47 (4)
C1···C3^iii^	3.4166 (19)		
			
C1—N1—C2	102.64 (11)	N3—C3—H3	117.3 (10)
C2—N2—N3	101.05 (10)	C3—C4—C5	120.13 (12)
C3—N3—N2	127.88 (11)	C3—C4—H4	120.6 (11)
C3—N3—C1	122.05 (11)	C5—C4—H4	119.3 (11)
N2—N3—C1	110.07 (11)	N4—C5—C4	122.68 (12)
C5—N4—C1	116.45 (11)	N4—C5—C6	117.78 (12)
N1—C1—N4	128.43 (12)	C4—C5—C6	119.54 (12)
N1—C1—N3	109.26 (11)	C5—C6—H6A	112.4 (14)
N4—C1—N3	122.30 (12)	C5—C6—H6B	111.1 (13)
N2—C2—N1	116.97 (12)	H6A—C6—H6B	106.4 (19)
N2—C2—H2	122.2 (10)	C5—C6—H6C	112.2 (14)
N1—C2—H2	120.8 (10)	H6A—C6—H6C	111.5 (19)
C4—C3—N3	116.38 (12)	H6B—C6—H6C	103 (2)
C4—C3—H3	126.3 (10)		
			
C2—N2—N3—C3	179.68 (13)	N3—N2—C2—N1	0.14 (17)
C2—N2—N3—C1	0.03 (14)	C1—N1—C2—N2	−0.24 (17)
C2—N1—C1—N4	179.97 (13)	N2—N3—C3—C4	−179.91 (12)
C2—N1—C1—N3	0.24 (14)	C1—N3—C3—C4	−0.30 (18)
C5—N4—C1—N1	−179.67 (12)	N3—C3—C4—C5	−0.18 (19)
C5—N4—C1—N3	0.04 (17)	C1—N4—C5—C4	−0.53 (17)
C3—N3—C1—N1	−179.85 (12)	C1—N4—C5—C6	178.84 (11)
N2—N3—C1—N1	−0.18 (14)	C3—C4—C5—N4	0.6 (2)
C3—N3—C1—N4	0.39 (18)	C3—C4—C5—C6	−178.74 (13)
N2—N3—C1—N4	−179.93 (11)		

Symmetry codes: (i) −*x*+2, −*y*+1, −*z*+2; (ii) *x*−1, *y*, *z*; (iii) *x*+1, *y*, *z*; (iv) *x*, −*y*+1/2, *z*+1/2; (v) *x*+1, −*y*+1/2, *z*+1/2; (vi) −*x*, −*y*+1, −*z*+1; (vii) −*x*+1, *y*+1/2, −*z*+3/2.

### Hydrogen-bond geometry (Å, º)

**Table:**

*D*—H···*A*	*D*—H	H···*A*	*D*···*A*	*D*—H···*A*
C2—H2···N1^i^	1.016 (17)	2.550 (19)	3.4052 (18)	141.5 (13)
C3—H3···N2^vi^	0.979 (18)	2.525 (18)	3.4822 (18)	165.8 (13)
C4—H4···N4^viii^	0.946 (19)	2.642 (19)	3.5677 (17)	165.9 (14)

Symmetry codes: (i) −*x*+2, −*y*+1, −*z*+2; (vi) −*x*, −*y*+1, −*z*+1; (viii) *x*−1, −*y*+1/2, *z*−1/2.
